# Traditional health practices in child care: Perceptions of caregivers in a township community

**DOI:** 10.4102/jphia.v17i1.1578

**Published:** 2026-02-27

**Authors:** Eugene M. Makhavhu

**Affiliations:** 1 Nursing Science Department, School of Healthcare Sciences, Sefako Makgatho Health Sciences University, Tshwane, South Africa

**Keywords:** alternative medicines, child healthcare, complementary medicine, indigenous health, traditional health practices

## Abstract

**Background:**

For women of childbearing age in South Africa, using different traditional healthcare practices is common. The use is intended for differing reasons, including aiding with conception and care of the baby during the neonatal phase and of the mother during the nursing stages. This is particularly so in underserved communities; however, perceptions of those who use such treatments are not widely explored.

**Aim:**

This study explored the perceptions of caregivers in township communities regarding traditional practices in healthcare.

**Setting:**

This study was conducted in a township in a metropolitan city in Gauteng.

**Methods:**

The study used a qualitative exploratory design, and data were collected using a semi-structured interview guide from 15 participants who were sampled conveniently.

**Results:**

Three themes were generated: (1) critical perspectives on traditional healthcare practices use, (2) benefits of using traditional healthcare and (3) the preference and influence on continued traditional healthcare use. Results indicated continued traditional healthcare practice use because of the belief systems and trust enshrined in individual families and communities. Benefits such as the ability to use multiple healthcare modalities were also a strong indication.

**Conclusion:**

This study highlights a strong need to understand the traditional healthcare practices to foster safe and comprehensive healthcare delivery and meet the health needs of communities.

**Contribution:**

The study contributes to literature on the use of traditional health practices and has captured the perceptions of caregivers in a South African township.

## Introduction

The realisation of inclusive access to healthcare is an important aspect of the healthcare delivery system, particularly in South Africa; however, for some contexts, that realisation may be difficult without the inclusion of alternative medicines. The Alma Ata declaration of 1978 posited that to have effective primary healthcare, it must rely on several other services, including traditional healthcare practices as needed.^1^ In South Africa, complementary and alternative medicines include the indigenous traditional healthcare practices that are offered by traditional healthcare practitioners, including healers, diviners, herbalists, traditional surgeons and traditional birth attendants, as recognised by the *Traditional Health Practitioner’s Act 22 of 2007*.^2^ The availability of such services at the lowest point in communities, including their affordability, relatability to the healthcare provider and a reduced travel cost, makes their utilisation relatively easy and continuous.

Statistics from a World Health Organization (WHO) 2023 survey indicated an exponential increase in the use of traditional health practices.^3^ Before the advent and domination of Western medicines, traditional healthcare practices were a big part of healthcare and were considered effective.^4^ The practices and health remedies have been an integral part of healthcare, and their use has a long history of cultural importance in South Africa^5^ and are thus embedded in people’s cultural beliefs. They are still immensely used in South Africa and are more commonly used in child healthcare for several healthcare needs, including spiritual and cultural interventions. Traditional healthcare practices have a role in child health even in modern society, particularly for families that are socialised around cultural and traditional activities such as protection of the pregnancy, child-birth, naming ceremonies, and other traditional activities that may be deemed as important for the cultural group.^6^

South Africa, with its multiple and diverse African cultural groupings including the Venda and Tsonga people, the Bapedi, Basotho, Batswana people, and the Nguni tribes which comprise the Zulu, Swati, Xhosa and Ndebele people, all have different child healthcare ceremonies followed and performed for various traditional occasions in various settings. Although different, all are rooted in culture and the spiritual beliefs of the families and are all used for a myriad of reasons, including accessibility, confidentiality and long-built trust.^7^ Traditional healthcare practices are also considered by many as a level of entry in the healthcare delivery system in Africa.^8^

For many communities and families, traditional healthcare practices are the only source of healthcare services, and as such remain relevant for those who make use of them. They are, in most cases, a part of the heritage of many Africans.^9^ Given that about 80% of people make use of traditional healthcare practices, for most South Africans in township settings with limited access to Western healthcare, traditional healthcare services remain their first line of access to healthcare, and hence their continued relevance. It is therefore important to acknowledge and protect the users of these services and at the same time understand their perceptions regarding traditional healthcare practices and their relevance, particularly in child healthcare.

## Research methods and design

### Study design

This study was qualitative^10^ in approach and followed an exploratory and descriptive design to enable the researcher a more complete understanding of the participants’ statements.^11^ The design was relevant to ensure an investigation of in-depth and narrative data. This was applied to investigate the caregivers’ perception relating to traditional healthcare practices in child health within their settings.

### Study setting

The study was conducted in a township north of a metropolitan city in Gauteng province, South Africa. The township has several clinics and a community healthcare centre catering for the population and serving a range of healthcare services, including the integrated management of childhood illnesses (IMCI) and immunisation services guided by the expanded programme on immunisation (EPI). There are several private healthcare facilities, including private nurse-led clinics and a private hospital that caters for community members that can afford the use of such services. The township is dominated by black Africans of multiple cultural backgrounds and beliefs, hence a thriving traditional health and spiritual practice industry where some community members consult for several healthcare needs, including child healthcare.

### Study population and sampling

The population in this study comprised children’s caregivers in their capacity as biological parents or close relatives of children who were direct caregivers and/or legal guardians. The participants in this study were sampled using convenience sampling, which is a procedure involving the selection of the most readily available people or objects for a study.^12^ The final sample size in this study comprised 15 participants and was determined by data saturation when newly collected data began to be the same as what had already been collected.^11^

### Data collection

Data were collected from participants using a semi-structured interview guide through individual in-depth interviews, which are data collection methods that seek people’s detailed description.^13^ The interviews were conducted by the author in English for participants who could respond and were comfortable with the language. Setswana, which is a dominant native language in the community, was also used for participants who were not conversant or were uncomfortable with the English language. A translation of the interview guide was done by the author prior to data collection to ensure consistency with data collection by correctly capturing the central and probing questions. An audio recorder was used to record the interviews, and participants were informed and gave consent for the recordings. The interviews lasted between 35 min and 55 min. Data were collected until the point of saturation, and the total sample size was realised at the 11th participant, with four more interviews conducted to confirm the saturation.

### Data analysis

Thematic analysis^14,15^ was used to analyse data. All recorded interviews were listened to and transcribed verbatim by the author. The analysis of the data entailed the reading of the transcripts to familiarise the author with the data and generate initial codes. From the data, the author searched for themes and made a review of potential themes, defining and naming them and finally writing up the report from the emerged themes. The researcher coded and gathered recurrent statements from the participants’ responses to give meaning to the data. The recurrent statements were then categorised as themes. An independent coder who had a Master’s degree and had 9 years of experience was appointed to analyse the data independently and formulate themes and subthemes following the analysis steps. A consensus discussion was arranged with the author and an independent co-coder to discuss and finalise the themes. Three themes were therefore agreed upon.

### Trustworthiness

Lincoln and Guba’s strategies to enhance trustworthiness by adhering to the principles of credibility, confirmability, dependability and transferability were followed in this study.^16^ To ensure credibility, standards, as set out in the study protocol, were adhered to, and there was no deviation from the study. Participants were interviewed in a language they could understand and were comfortable with to ensure consistency. There was a clear criterion set for participation, and the study followed that. To capture participants’ responses thoroughly, paraphrasing and question probing during interviews were used to enhance member checking. An audit trail of study data, including interview transcripts and interview recordings, is kept and maintained to ensure dependability. Direct quotes from participants were used to ensure that the collected data accurately reflect participants’ responses and to enhance confirmability while preventing bias.

### Ethical considerations

The study received ethical clearance and was approved by the Tshwane University of Technology Ethics Review Committee (FCRE 2020/05/003 [FCPS02][SCI]). Furthermore, permission was granted by the district health authority in the metropolitan municipality (GP_202006_020). All participants who took part in the study provided written informed consent and were made aware of their rights throughout the study period. Ethical standards of research were followed, including privacy, confidentiality and beneficence and non-maleficence. Interviews were handled privately, and the personal details were not collected to ensure that privacy is maintained. Confidentiality was ensured by coding all interview recordings and transcripts with a number instead of using participants’ potential identifiers. The study anticipated no harm to the participants; however, they were informed of their ability to cease participation in the unlikely event of discomfort.

## Results

### Sociodemographic data

The study comprised a total of 15 caregivers. Of the 15 caregivers, two were men, while 13 were women. The population of caregivers included biological parents and caregivers in their roles as grandparents or other close relatives with healthcare decision-making powers for the children under their care. The participants’ ages ranged between 19 years and 50 years, and the majority of them were unemployed at the time of data collection (Table 1).

**TABLE 1:** Demographic data.

Participants’ code	Participants’ age	Participants’ gender	Employment status
Participant 1	27 years old	Female	Permanently employed
Participant 2	33 years old	Male	Permanently employed
Participant 3	40 years old	Male	Unemployed
Participant 4	20 years old	Female	Unemployed
Participant 5	50 years old	Female	Permanently employed
Participant 6	22 years old	Female	Unemployed
Participant 7	34 years old	Female	Permanently employed
Participant 8	23 years old	Female	Unemployed
Participant 9	35 years old	Female	Permanently employed
Participant 10	28 years old	Female	Unemployed
Participant 11	22 years old	Female	Unemployed
Participant 12	38 years old	Female	Unemployed
Participant 13	21 years old	Female	Unemployed
Participant 14	19 years old	Female	Unemployed
Participant 15	35 years old	Female	Unemployed

### Overview of themes

Three main themes were generated from this study, supported by 7 subthemes following the analysis of data. The themes are presented in Table 2.

**TABLE 2:** Themes and subthemes.

Theme	Subtheme
1. Critical perspectives on the use of traditional healthcare practices	1.1 Non-believer in traditional healthcare 1.2 Discomfort and fear of the unknown
2. Benefits of using traditional healthcare for health needs	2.1 Efficacy of traditional practices 2.2 An opportunity to complement healing modalities in healthcare
3. Preference versus influence on the use of traditional healthcare	3.1 Cultural, spiritual beliefs and significance 3.2 Family influence on traditional healthcare 3.3 Traditional healthcare’s success in the treatment of conditions

#### Theme 1: Critical perspectives on the use of traditional healthcare practices

Two subthemes emerged from this theme, namely non-believer in traditional healthcare and discomfort and fear of the unknown.

**Subtheme 1.1: Non-believer in traditional healthcare:** Some of the caregivers gave their perspectives relating to traditional healthcare practices and cited not believing in their efficacy. This indicated their scepticism about using traditional healthcare practices, and that was centred around their beliefs:

‘I just don’t believe in them [*traditional health practices*].’ (Participant 1, Female, 27 years old)

‘I don’t believe in traditional healthcare practices. I would rather take my children to church for any non-medical spiritual needs.’ (Participant 12, Female, 38 years old)

**Subtheme 1.2: Discomfort and fear of the unknown:** The participants also highlighted their discomfort and fears relating to traditional healthcare practices. The fear was mostly linked with not knowing how the child would react to the medicinal herb that is applied or consumed, as the content of the herb is kept a secret:

‘Seeing my child being cut to apply whatever medicine … it was really hard and scary.’ (Participant 1, Female, 27 years old)

‘You don’t know what danger it poses to the child or what substances were used in that medicine.’ (Participant 3, Male, 40 years old)

‘I don’t even know what is it that they have put in there nor the dangers of the medicine.’ (Participant 12, Female, 38 years old)

#### Theme 2: Benefits of using traditional healthcare for health needs

Two subthemes emerged where participants narrated their perceptions on the benefits of traditional healthcare practices. The efficacy of traditional practices and the opportunity for users to complement healthcare were highlighted.

**Subtheme 2.1: Efficacy of traditional practices:** Although some participants cited not believing in traditional healthcare and their fears towards it, some of the participants in this study highlighted that traditional health practices are beneficial and effective for some ailments in the traditional realm. In some cases, such ailments are believed to be difficult to treat using allopathic healthcare practices:

‘Sometimes the clinics or hospitals may not be able to see the cause of the illness and the traditional healers may be able to.’ (Participant 2, Male, 33 years old)

‘I know that they help a child when the child is still young. They strengthen the child and treat them for Hlogwana and worms.’ (Participant 14, Female, 19 years old)

‘You know there are like some diseases that the clinic may not be able to diagnose or treat. So maybe a traditional haler can be able to note and treat that condition.’ (Participant 9, Female, 35 years old)

‘The indigenous-traditional health practitioners are the only people who can assist with such treatments of Hlogwana, Sefola and the likes.’ (Participant 13, Female, 21 years old)

**Subtheme 2.2: An opportunity to complement healing modalities in healthcare:** The use of traditional health practices was seen as an opportunity to make use of complementary medicines in the healthcare space. The participants indicated that if the services they received from the hospital or clinic were not sufficient, then the traditional healthcare practices presented an opportunity to try another line of treatment:

‘… sometimes in the clinic when you bring a child with certain health complications they just give you Pain pills … but the herbs the traditional health practitioner give or mix for you can help the child better.’ (Participant 11, Female, 22 years old)

‘If it is a very serious condition and the hospital is telling me that they can’t help my child or that they can’t see anything. I will have to go there [*to a traditional health practitioner*] for my baby’s sake.’ (Participant 15, Female, 35 years old)

‘If my child is really sick and I am not getting help in the clinics and the hospitals, I will definitely stand up and seek help anywhere including at a traditional healer just so my child can get help and stop suffering. So I would do it for the sake of the child.’ (Participant 4, Female, 20 years old)

Another participant emphasised the need for spiritual healing as another reason to attend or seek intervention from a traditional healthcare practitioner:

‘I think it is right, and very important because if a person comes to the clinic and get treatment, I believe sometimes our bodies still need the spiritual healing and protection [*from a traditional healer*].’ (Participant 5, Female, 50 years old)

#### Theme 3: Preference versus influence on the use of traditional healthcare

This theme presented three subthemes that focused on the influence of continued use of traditional healthcare practices.

**Subtheme 3.1: Cultural, spiritual beliefs and significance:** The participants in this study indicated that the continued use of traditional healthcare practices is centred around the cultural and spiritual beliefs of people. Ancestral rituals were also cited by participants as one other significance of traditional healthcare practices:

‘People believe that they will get healing. It could be things related to spiritual healing or ancestral calling, hence the continued use of traditional health practices.’ (Participant 1, Female, 27 years old)

‘Sometimes maybe a person just needs to cleanse or acknowledge ancestors or even perform certain rituals, not necessarily that they are sick.’ (Participant 5, Female, 50 years old)

‘They believe that they will get healing for prolonged illnesses, basically protection against evil spirit.’ (Participant 11, Female, 22 years old)

‘They [*traditional healthcare practices*] help strengthen the baby against any evil spirits.’ (Participant 6, Female, 22 years old)

**Subtheme 3.2: Family influence on traditional healthcare:** Family influence was also quoted as a stronghold to the continued traditional healthcare practices use and ensuring their continued relevance in healthcare-seeking behaviours of African communities. The importance of culture and family background in participants’ health-seeking behaviour was also indicated by participants:

‘My sister does them [*traditional healthcare practices*] for us … and for all our grandchildren as well.’ (Participant 5, Female, 50 years old)

‘My parents advised me that from our upbringing they made use of such.’ (Participant 8, Female, 23 years old)

‘My grandmother told me that it was spiritual [*child’s health problem*] but she still refused to go. At the end, my grandmother took her to the healer and she got healing.’ (Participant 10, Female, 28 years old)

‘… My aunt advised her to take her sick child to a healer and she decided to do so …’ (Participant 7, Female, 34 years old)

**Subtheme 3.3: Traditional healthcare’s success in treatment of conditions:** The participants cited cases where traditional healthcare practices were successful in healing children from an assortment of ailments. The successful treatment of those conditions in the past was seen as influential in the continued use and relevance of traditional healthcare practices:

‘… that same evening after being seen by the traditional healer, she [*daughter*] slept okay and was not crying at all.’ (Participant 14, Female, 19 years old)

‘I was suffering from something called “Kgetlhane” so then they did all the practices including strengthening as they would do on a child as it was not done for me when I was younger … After that, I was fine and back to my normal self.’ (Participant 8, Female, 23 years old)

‘A cousin of mine also did that [*took their child to a traditional healer*]. Her three months old child had a sunken fontanel and was not looking good. She went to the clinic, they gave her motskwako [*oral rehydration therapy*] and inserted a drip, it didn’t work. She was advised her to take the child to a sangoma [*diviner*] and she did. I don’t know what the sangoma did but the child was healed in two days. She was sort of awake, playing and looking like a normal child.’ (Participant 11, Caregiver, 22 years old)

## Discussion

This study’s findings presented an understanding of the continued relevance of traditional healthcare practices in child health in the modern era from the perspectives of the caregivers and guardians, particularly in underserved communities where healthcare service limitations are experienced, such as in the context of this study. This highlights the importance of a culturally sound and sensitive healthcare approach in order to meet the ever-growing healthcare demands of communities.^17^ The findings further revealed that some participants did not believe in traditional healthcare systems. This can be seen as a lack of trust in the ways or methods followed by traditional healthcare practitioners. One study implied that building trust between patients and healthcare providers is crucial for reducing disparities and improving health outcomes across diverse populations.^18^ However, the findings from this study indicated scepticism accompanied by fear and a lack of trust in traditional healthcare practices among some caregivers. This finding is in contrast to another study that found that the distrust in healthcare practices was aimed at allopathic or conventional healthcare practices and the decline in trust was correlated with an increased use of complementary and alternative medicine.^19^

Furthermore, the findings presented a fear of the unknown regarding traditional healthcare practices. The proliferation of traditional healthcare practitioners in South Africa also raises questions of the authenticity of practitioners, which also brings forth questions about the safety of their offerings, hence the fears from users and potential users regardless of their easy access in communities. This is further exacerbated by the mushrooming of imposters posing as traditional healthcare practitioners, as found in Chateau’s study, where participants expressed concern over devious imposters posing as traditional healthcare practitioners and giving a bad reputation to African traditional health practices.^20^ The fear and scepticism about the safety of traditional health practices lead to mistrust of services offered by traditional healthcare practitioners. This can be attributed to the lack of scientific backing of traditional healthcare practices, which further hinders the safe integration of traditional and allopathic healthcare.^21^

Despite the fears and mistrust cited by some participants, others highlighted the advantages of traditional healthcare practices for ailments that were thought to be better managed using traditional methods of treatment. This finding is similar to that of a study in India that posits that some traditional Indian healthcare practices were gaining recognition because of their benefits in treating various ailments.^22^ The findings revealed a general notion from the majority of participants that traditional healthcare practices were beneficial.

The findings of this study presented the perceived efficacy of traditional healthcare practices as one reason for the continued relevance and trusted use of traditional healthcare practices. This is supported by one study which stated that despite challenges, traditional healthcare practices remain popular in some regions in Nigeria because of factors such as their accessibility, cost and efficacy.^3^ Similarly, in a different study, the participants agreed that in their experience, complementary medicines, which included herbal medicines, worked well when used with conventional medicines for the treatment of infantile colic.^23^ Similarly, studies conducted in Africa focusing on traditional healthcare practices indicate continued use of different traditional healthcare practitioners and services because of their health benefits, just as this study found. Similarly, a 2023 study found a common view that traditional birth attendants were found to be more effective in terms of managing deliveries and postnatal complications that may arise.^24^

The study findings also suggest the influence of family and culture in caregivers making the decision to use traditional healthcare practices. This is supported by a study that found that people used traditional methods of disease treatment because they are entrenched in the cultural heritage, identity and their customs.^7^ Similarly, it was highlighted in another study that traditional medicines were deeply ingrained in the cultural heritage of the participants, which represents a legacy that is passed through generations within families and communities.^25^ Thipanyane et al. also posit that family members played a critical role in sharing information about the use of traditional healthcare practices, which is a finding similar to this study.^26^ This places the importance of family as central in spiritual and traditional healing, as highlighted in another study that there is great importance placed on the family and the need to obtain a family history before calling on the ancestors, and further emphasis is placed on seeking consent from the elders before certain rituals are performed or certain traditional medications are given.^20^ This finding is similar to those of a study conducted in Zambia, which found that the participants reported feeling pressured by family members and the elderly in their families to make use of traditional healthcare practices during pregnancy.^27^ This family influence is generational, just as traditional healthcare practices and cultural rituals are passed on from one generation to the next; thus, traditional healthcare practices will maintain their relevance in African healthcare-seeking behaviours as they are passed on through generations.

Moreover, the findings also suggested the significance of culture as influential to participants’ continued use and maintaining the relevance of traditional healthcare practices. This is supported by a Sudanese study whose findings cited that cultural influence was one of the reasons for seeking traditional medicine, as cited by 57% of the respondents in that study.^28^ This study refers to the relevance of traditional healthcare practices as being closely linked to the beliefs of healing, as cited by the participants. This is similar to another study that posited that the views of the participants about traditional medicines appeared to be intertwined with their beliefs in the aetiology of diseases.^25^

Additionally, this study found that the participants believed in the successful treatment of certain conditions using traditional healthcare practices, a finding supported by another study where participants did not seek allopathic healthcare services, thus citing traditional medicines as being more effective to address such.^25^

### Strengths and limitations

This study followed a qualitative research approach, which allowed for the collection of rich narrative data to obtain details of participants’ perspectives on traditional healthcare. Participants’ exact perceptions coupled with their emotions were rightly captured by using interviews.

Although bias was minimised and participants reassured, sampling of participants in allopathic healthcare facilities may have limited the freedom of responses from some participants; therefore, for future research, participants may be sampled from different settings in communities where they will be free to answer questions without the potential fear of limited healthcare services provision.

## Conclusion

The extent to which traditional healthcare practices remain available and easily accessible to township communities is the very reason for their continued relevance. To ensure healthcare delivery that is not limited to the privileged, it is important to acknowledge the use of traditional healthcare services by those who deem them effective. This study concludes that healthcare is a basic need and therefore may not be limited to a select few in terms of accessibility; however, it is also important to note that the safe use of traditional healthcare practices and medicines for whatever reasons may be an important aspect of maintaining healthy communities. In order to ensure universal coverage, the traditional healthcare practitioner as the first point of contact and, in some instances, the only healthcare provider for some communities cannot be left behind and negated.

## References

[1] International Conference on Primary Health Care. Alma Ata declaration. Geneva: WHO; 1978.

[2] Republic of South Africa. Traditional Health Practitioners Act, No. 22 of 2007. Pretoria: Government Printers; 2007.

[3] Ibrahim MA, Olaitan AA. Traditional healthcare practices: Growing demands and emerging trends. GSC Adv Res Rev. 2022;13(2):69–79. 10.30574/gscarr.2022.13.2.0296

[4] Sunday AC, Chukwuma OJ. Traditional medicine in the face of new era: A better safeguarding for the progression of healthcare claim in Igbo-African world. J Afr Stud and Sust Dev. 2021;4(4).

[5] Williams VL, Whiting MJ. A picture of health? Animal use and the Faraday traditional medicine market, South Africa. J Ethnopharmacol. 2016:179:265–273. 10.1016/j.jep.2015.12.024 26727647

[6] Makhavhu EM, Masala-Chokwe ME, Ramukumba TS. Traditional health-seeking behaviour of children’s caregivers in a township in the City of Tshwane, South Africa. Afr J Phys Act Health Sci. 2023;29(1):37–51. 10.37597/ajphes.2023.29.1.3

[7] Mutola S, Pemunta NV, Ngo NV. Utilization of traditional medicines and its integration into the healthcare system in Qolokweni, South Africa; prospects for enhanced universal coverage. Complement Ther Clin Pract. 2021;43:101386. 10.1016/j.ctcp.2021.101386 33895465

[8] Thipanyane MP, Nomatshila SC, Musarurwa HT, Oladimeji O. The roles and challenges of traditional health practitioners in maternal health services in rural communities of Mthatha, South Africa. Int J Environ Res Public Health. 2022;19(20):13597. 10.3390/ijerph192013597 36294175 PMC9603220

[9] Booth Z. Traditional medicines should be used in healthcare [homepage on the Internet]. University of Witwatersrand. 2023 [cited 2025 May 12]. Available from: https://www.wits.ac.za/news/latest-news/opinion/2023/2023-08/traditional-medicines-should-be-used-in-healthcare.html.

[10] Polit DF, Beck CT. Nursing research: Generating and assessing evidence for nursing practice. 11th ed. Philadelphia, PA: Wolters Kluwer; 2021.

[11] Grove SK, Gray JR. Understanding nursing research: Building an evidence-based practice. 7th ed. St Louis, MO: Elsevier; 2019.

[12] Brink H, Van der Walt C, Van Rensburg G. Fundamentals of research methodology for healthcare professionals. 4th ed. Cape Town: Juta; 2018.

[13] Spickard J. Research basics: Design to data analysis in 6 steps. California: Sage; 2017.

[14] Ahmed SK, Mohammed RA, Nashwan AJ, et al. Using thematic analysis in qualitative research. J Med Surg Public Health. 2025;6:100198. 10.1016/j.glmedi.2025.100198

[15] Braun V, Clarke V. Using thematic analysis in psychology. Qual Res Psycho. 2006;3(2):77–101. 10.1191/1478088706qp063oa

[16] Lincoln YS, Guba EG. Naturalistic inquiry. Newbury Park, CA: Sage; 1985.

[17] Makhavhu EM. Integrating traditional and allopathic child health: A healthcare transformation opportunity. Health SA Gesondh. 2024;29:a2501. 10.4102/hsag.v29i0.2501 PMC11079414 38726057

[18] Khullar D, Darien G, Ness DL. Patient consumerism, healing relationships, and rebuilding trust in healthcare. J Am Med Assoc. 2020;324(23):2373–2374. 10.1001/jama.2020.12938 33320232

[19] Barbieri V, Lombardo S, Gartner T, Piccoliori G, Engl A, Wiedermann CJ. Trust in conventional healthcare and utilization of complementary and alternative medicine in South Tyrol, Italy: A population-based cross-sectional survey. Ann Ig. 2024;36(4):377–391. 10.7416/ai.2024.2605 38386023

[20] Chateau AV, Gqaleni N, Aldous C, Dloca N, Blackbeard D. A qualitative study on traditional healers’ perceptions and management of epidermolysis bullosa. Health SA Gesondh. 2023;28:2266. 10.4102/hsag.v28i0.2266 PMC10476505 37670748

[21] Pal A, Santra A, Panigrahi S. Broadening the avenues of complementary medicine and way forward to integrate it with evidence-based modern medicine research – A need of the hour. Natl J Indian Assoc Prev Soc Med. 2025;37(2):183–188. 10.47203/IJCH.2025.v37i02.002

[22] Kareem AA, Yoganandham G. A study of the traditional health care practices in ancient Tamil Nadu – An assessment. Int J Emerg Res Eng Sci Manag. 2022;1(3):7–10. 10.58482/ijeresm.v1i3.2

[23] Di Gaspero NC, Razlog R, Patel R, Pellow J. Perceived effectiveness of complementary medicine by mothers of infants with colic in Gauteng. Health SA Gesondh. 2019;24:a1175. 10.4102/hsag.v24i0.1175 PMC6917380 31934429

[24] Dektar B, Beckford AN, Kemba J, Crayson B. Mothers’ experiences and perceptions about care provided during home deliveries in Alwa sub county, Kaberamaido district, Uganda – A qualitative study. Front Public Health. 2023;11:1180945. 10.3389/fpubh.2023.1180945 37920578 PMC10619897

[25] Fauk NK, Seran AL, Asa GA, et al. Parental experiences and perceptions of using traditional medicine and biomedical services for their children’s health: A qualitative study in Indonesia. Res Sq. 2024. 10.21203/rs.3.rs-4700136/v1

[26] Thipanyane MP, Nomatshila SC, Oladimeji O, Musarurwa HT. Perceptions of pregnant women on traditional health practices in a rural setting in South Africa. Int J Environ Res Public Health. 2022;19(7): 4189. 10.3390/ijerph19074189 35409871 PMC8998603

[27] El Hajj M, Sitali DC, Vwalika B, Holst L. ‘Back to Eden’: An exploratory qualitative study on traditional medicine use during pregnancy among selected women in Lusaka Province, Zambia. Complement Ther Clin Pract. 2020;40:101225. 10.1016/j.ctcp.2020.101225 32798811

[28] Ahmed GEM, Ahmed EYM, Ahmed AE, et al. Prevalence and reasons to seek traditional healing methods among residents of two localities in North Kordofan State, Sudan 2022: A cross-sectional study. Health Sci Rep. 2023;6(8):e1487. 10.1002/hsr2.1487 37621385 PMC10444970

